# Design and Measurement of Microelectromechanical Three-Axis Magnetic Field Sensors Based on the CMOS Technique

**DOI:** 10.3390/mi14051038

**Published:** 2023-05-12

**Authors:** Chi-Han Wu, Cheng-Chih Hsu, Yao-Chuan Tsai, Chi-Yuan Lee, Ching-Liang Dai

**Affiliations:** 1 Department of Mechanical Engineering, National Chung Hsing University, Taichung 402, Taiwan; 2 Department of Electro-Optical Engineering, National United University, Miaoli 360, Taiwan; 3 Department of Bio-Industrial Mechatronics Engineering, National Chung Hsing University, Taichung 402, Taiwan; 4 Department of Mechanical Engineering, Yuan Ze Fuel Cell Center, Yuan Ze University, Taoyuan 320, Taiwan

**Keywords:** three-axis magnetic field sensor, cross-sensitivity, sensitivity, MEMS, CMOS

## Abstract

The design, fabrication, and measurement of a microelectromechanical system (MEMS) three-axis magnetic field sensor (MFS) based on the commercial complementary metal oxide semiconductor (CMOS) process are investigated. The MFS is a magnetic transistor type. The performance of the MFS was analyzed employing the semiconductor simulation software, Sentaurus TCAD. In order to decrease the cross-sensitivity of the three-axis MFS, the structure of the MFS is planed to accommodate two independent sensing components, a z-MFS utilized to sense magnetic field (M-F) in the z-direction and a y/x-MFS composed of a y-MFS and a x-MFS to be utilized to sense M-F in the y- and x-directions. The z-MFS incorporates four additional collectors to increase its sensitivity. The commercial 1P6M 0.18 μm CMOS process of the Taiwan Semiconductor Manufacturing Company (TSMC) is utilized to manufacture the MFS. Experiments depict that the MFS has a low cross-sensitivity of less than 3%. The sensitivities of z-, y-, and x-MFS are 237 mV/T, 485 mV/T, and 484 mV/T, respectively.

## 1. Introduction

In electronics, industrial equipment and biomedical engineering, magnetic field sensors (MFSs) represent an important sensing component and are utilized to measure the magnetic field (M-F). MFSs have various applications. For instance, Juarez-Aguirre [1] utilized a microelectromechanical system (MEMS) MFS, a virtual instrument, and a data acquisition card to construct a signal processing system for biomedical application. The signal processing system could sense magnetic flux density, and the system was actually used to detect magnetic flux density in a rat thoracic cage. A respiratory training system, proposed by Kang [2], was applied to monitor patient respiration. The system contained a MEMS patch-type MFS that could measure the change of magnetic intensity corresponding to the movement of respiration for the thoracic surface of patients. Lu [3] presented a location tracking system with MEMS MFSs for clinical application. The system could track the organ and vessel shape of a patient through the MS node sensor. Feng [4] developed a microfluidic platform with a giant magnetoimpedance MFS for biomarker detection. The MFS was used to sense the magnetic beads in a microfluidic system. Lara-Castro [5] proposed a portable signal conditioning system with a resonant MEMS MFS for industrial application. The portable system could sense the residual M-F for ferromagnetic materials. Fourati [6] employed a three-axis Hall MFS, an accelerometer, and a gyroscope to develop a system, which was used in bio-logging to detect the dynamic body acceleration estimation. A nonlinear filter evaluated the sensing signals of the sensor in the system. Xu [7] used a Hall MFS, an angular rate sensor, and a gravity sensor to make an attitude estimator that adopted the Kalman filter for estimating the attitude. The flight information measuring device with a MEMS MFS, proposed by Ma [8], was used in aircraft navigation. The measuring device could detect the flight attitude of aircraft. 

Three-axis magnetic field sensors are able to measure magnetic fields in three directions simultaneously. In practical applications, magnetic fields are often not unidirectional, so it is necessary to measure magnetic fields in all directions to obtain more comprehensive information. For example, in applications, such as magnetic field navigation and positioning, magnetic field imaging, and Earth magnetic field measurement, it is necessary to measure magnetic field information in multiple directions. Therefore, this study develops a magnetic field sensor that can simultaneously measure magnetic fields in three axes. 

The MEMS technology used in microfabrication and micromachining processes was utilized to develop microactuators [9,10,11,12,13] and microsensors [14,15,16,17,18]. Many studies presented the use of the MEMS technology in developing various MFSs. For example, Du [19] developed a one-axis resonant MFS and the structure of the MFS was composed of dual-coupled photonic crystal cavities with two nanobeams. The nanobeams were driven using Lorentz force. The MFS was made using the MEMS technology. The MFS sensitivity was 22.9 mV/T. The output signal of the sensor was easily influenced by vibration as the sensor was of a resonant type. Gkotsis [20] presented a one-axis resonant MFS and utilized the bulk micromachining to make the resonant MFS on a SOI (silicon on insulator) wafer. The resonant MFS was driven using Lorentz force and was sensed by piezoresistive element. The resonant MFS sensitivity was 513 mV/T. The output signal of the sensor was easily affected by vibration since the sensor was a resonant type. Tseng [21] employed the CMOS process to make a one-axis MFS. The structure of the MFS included the magnetic transistor. The MFS was integrated with circuitry, which was utilized to enhance the MFS output voltage (O-V). The MFS sensitivity was 354 mV/T. Herrera-May [22] used bulk micromachining to develop a one-axis resonant MFS. The structure of the resonant MFS contained a plate, an aluminum loop, two support beams, and four flexural beams. The resonant MFS was driven by Lorentz force. The driving voltage and power consumption of the MFS were 3 V and 12 mW, respectively, and the MFS sensitivity was 230 mV/T. Because the sensor was of a resonant type, the output signal of the sensor was easily influenced by vibration. Liu [23] developed a fluxgate MFS using the MEMS technology with the electroplating process. The structure of the fluxgate MFS had a closed double-layer core, which was made of Fe-based ribbon deposited by the electroplating process. The electroplating process for Fe-based ribbon was not compatible with the CMOS process. Zhao [24] designed a two-axis MFS and used the MEMS technology to make the MFS. The structure of the MFS was constructed by four magnetic transistors and four resistors. The sensitivities of the MFS were 223 mV/T in the x-axis M-F and 218 mV/T in the y-axis M-F. Yang [25] developed a two-axis MFS and employed the MEMS technology with packaging technique to make the MFS on a silicon wafer. The MFS contained two magnetic transistors, one that was set in the x-axis direction, which detected M-F in the x-axis, and the other set in the y-axis direction, which detected M-F in the y-axis. The sensitivities of the MFS were 366 mV/T in the x-axis M-F and 365 mV/T in the y-axis M-F. Zhao [26] presented a three-axis MFS that was made employing MEMS technology. The MFS consisted of a Hall sensor and four magnetic transistors. The sensitivities of the MFS in the x-, y-, and z-axis M-F were 77.5 mV/T, 78.6 mV/T, and 77.4 mV/T, respectively. Chen [27] proposed a three-axis MFS fabricated utilizing the CMOS process. The structure of the MFS contained two magnetic field effect transistors, which were utilized to improve the sensitivity of the MFS. The MFS sensitivity was 182 mV/T in the x-axis M-F. The MFS had a sensitivity of 180 mV/T in the y-axis M-F and a sensitivity of 27.8 mV/T in the z-axis M-F. Wu [28] used the CMOS process to design and manufacture a three-axis MFS. The MFS was composed of two magnetic sensing elements, one of which was utilized to sense M-F in the x- and y-axis and the other employed to sense M-F in the z-axis. The magnetic sensing elements consisted of the magnetic transistors. The MFS had a sensitivity of 119 mV/T in the z-axis M-F. The sensors of Du [19], Gkotsis [20], Tseng [21], Herrera-May [22], and Liu [23] were one-axis MFSs, and the sensors of Zhao [24] and Yang [25] were two-axis MFSs. The sensors of Zhao [26], Chen [27], and Wu [28] were three-axis MFSs. We designed and fabricated a three-axis MFS with higher sensitivity compared to the MFSs [19,20,21,22,23,24,25,26,27,28]. There are several types of magnetic sensors, such as Hall effect sensors, anisotropic magnetoresistive (AMR) sensors, giant magnetoresistive (GMR) sensors, and magnetic transistor sensors. Hall effect sensors can be produced by a simple manufacturing process and are low-cost, but their sensitivity is lower. AMR and GMR sensors have high sensitivity, but they require more complex processes and materials, resulting in higher costs. Magnetic transistor sensors have high gain and sensitivity, as well as the advantages of low cost, high process stability, and mass-production by semiconductor foundries. Hence, this study develops a magnetic transistor MFS.

Various micro components [29,30,31,32,33] and devices [34,35,36,37,38] are manufactured employing the CMOS process. Micro MFSs developed by this process have many benefits [27,28]. This study adopts this process to make a three-axis MEMS MFS. The MFS is composed of z-MFS and a y/x-MFS. The z-MFS is a magnetic transistor type with four additional collectors that enhance sensitivity. The y/x-MFS consists of a y-MFS and an x-MFS. The y-MFS and an x-MFS are utilized to sense M-F in the y- and x-direction, respectively.

## 2. Structure and Analysis of Magnetic Field Sensor

**Figure 1:**
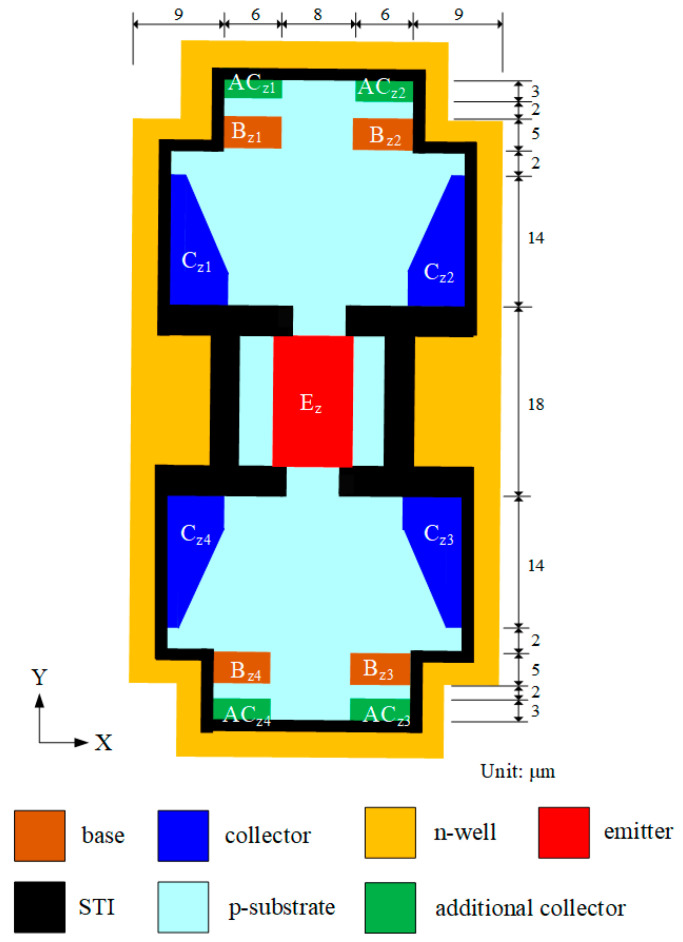
Schematic structure of z-magnetic field sensor (MFS).

The three-axis MFS chip contains an independent z-MFS and a y/x-MFS that is a combination of y-MFS and x-MFS. The x-MFS, y-MFS, and z-MFS are utilized to sense in the x-, y-, and z-axes M-F, respectively. Figure 1 illustrates structure of the z-MFS, where E_z_ is emitter; C_z1_, C_z2_, C_z3_, and C_z4_ are collector; B_z1_, B_z2_, B_z3,_ and B_z4_ are base; and AC_z1_, AC_z2_, AC_z3_, and AC_z4_ are additional collectors. The carriers flow from the emitter (E_z_) to the bases (B_z1_, B_z2_, B_z3_, and B_z4_), the additional collectors (AC_z1_, AC_z2_, AC_z3_ and AC_z4_) and collectors (C_z1_, C_z2_, C_z3_, and C_z4_) when the bias supplies the bases, collectors, and additional collectors. When a z-direction M-F applies to the z-MFS, a Lorentz force that is produced through the action of M-F and current acts on the carriers, which are deflected toward the collectors (C_z2_ and C_z4_) and the additional collectors (AC_z2_ and AC_z4_). The collectors of C_z2_ and C_z4_ have a higher current than the collectors of C_z1_ and C_z3_, such that there exists a of voltage difference between C_z1_ and C_z2_ and a voltage difference between C_z3_ and C_z4_. The O-V of the z-MFS is gained by the voltage differences in series.

**Figure 2:**
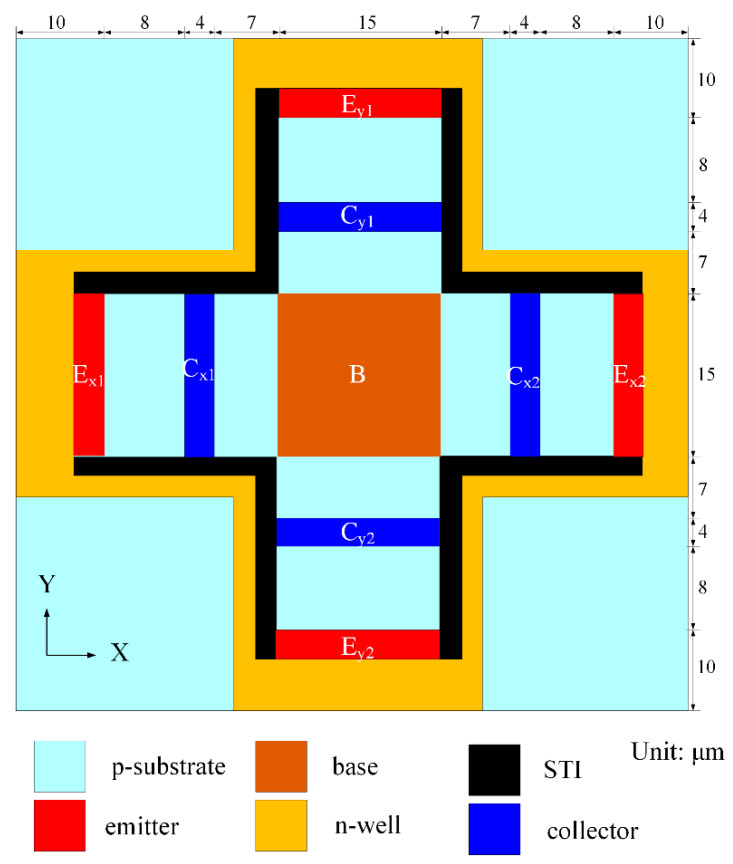
Schematic structure of y/x-MFS structure.

Figure 2 shows the schematic structure of the y/x-MFS, where B is base; C_x1_, C_x2_, C_y1_, and C_y2_ are collectors; E_x1_, E_x2_, E_y1_, and E_y2_ are emitters; and STI is shallow trench isolation. The y-MFS, which consists of a base (B), two collectors (C_x1_ and C_x2_), and two emitters (E_x1_ and E_x2_), is combined by two magnetic transistors. The shape between the x-MFS and the y-MFS are a symmetrically intersectional structure. The x-MFS, which consists of a base (B), two collectors (C_y1_ and C_y2_), and two emitters (E_y1_ and E_y2_), is also combined by two magnetic transistors.

As shown in Figure 2, the carriers flow from the emitters (E_x1_ and E_x2_) to the collectors (C_x1_ and C_x2_) and bases (B) when there are bias voltages supplied to the collectors (C_x1_ and C_x2_) and base (B). When a y-direction M-F applies to the y-MFS, a Lorentz force that is produced through the action of M-F and current acts on the carriers. The carriers are deflected upward the collector of C_x1_. On the other hand, the carriers are deflected downward the collector of C_x2_. The collector of C_x1_ has a higher current than the collector of C_x2_, such that there exists a voltage difference between C_x1_ and C_x2_. The O-V of the y-MFS is gained by voltage difference between C_x1_ and C_x2_. Similarly, the carriers flow from the emitters (E_y1_ and E_y2_) to the collectors (C_y1_ and C_y2_) and bases (B) when there are bias voltages supplied to the collectors (C_y1_ and C_y2_) and base (B). When an x-direction M-F applies to the x-MFS, a Lorentz force that is produced through the action of M-F and current acts on the carriers. The carriers are deflected upward the collector of C_y1_. On the other hand, the carriers are deflected downward the collector of C_y2_. The collector of C_y1_ has a higher current than the collector of C_y2_, such that there exists a voltage difference between C_y1_ and C_y2_. The O-V of the x-MFS is gained by a voltage difference between C_y1_ and C_y2_.

**Figure 3:**
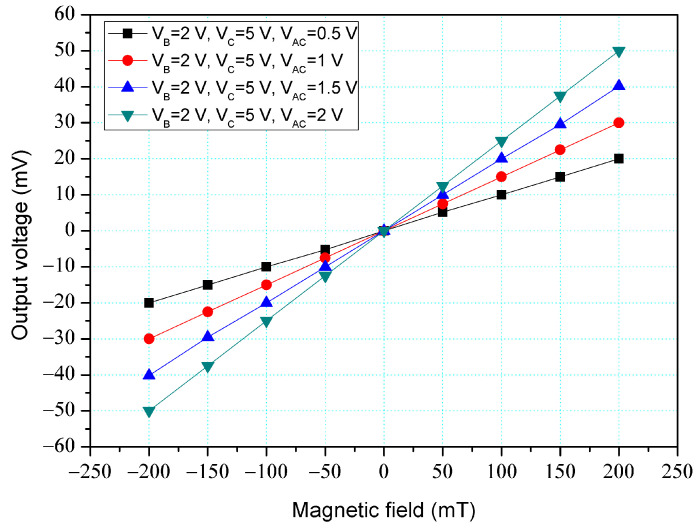
Output voltage (O-V) for z-MFS by simulation.

The simulation software, Sentaurus TCAD, was utilized to evaluate the characteristic of the z-MFS and y/x-MFS. The simulation steps involved establishing the model, mesh model, set material, and condition, selecting the relevant method, and carrying out computation [39]. According to the structures (Figure 1 and Figure 2), the z-MFS and y/x-MFS models were established respectively. The z-MFS and y/x-MFS models were meshed by the Delaunay triangulation approach. The coupling effect of M-F and electrical field was calculated by the Poisson electron hole method. The distribution of carrier density was evaluated by the Bank–Rose method. Figure 3 shows the O-V for the z-MFS by the simulation, where V_B_ is base bias; V_C_ is collector bias, and V_AC_ is additional collector bias. The additional collectors and the collectors were linked to a resistance of 1 kΩ, respectively. The collector bias and base bias were 5 V and 2 V, respectively. The different voltages of 0.5, 1, 1.5, and 2 V were inputted to the additional collectors. The results presented that the O-V for the z-MFS increased as the additional collector bias increased. When the collector, base, and additional collector bias were 5, 2, and 2 V, respectively, the O-V for the z-MFS was 50 mV at 200 mT. The use of the linear regression method fits the curve at V_B_ = 2 V, V_C_ = 5 V, and V_AC_ = 2 V. The regression line had a coefficient of determination R^2^ = 0.9999 and a slope of 250 mV/T. Therefore, the sensitivity of the z-MSE was 250 mV/T at V_B_ = 2 V, V_C_ = 5 V, and V_AC_ = 2 V, and the output linearity was 99%. The optimal operating point setting for z-MFS was V_B_ = 2 V, V_C_ = 5 V, and V_AC_ = 2 V, and with the load resistance of 1 K. As shown in Figure 3, the output voltage of the z-MFS increases as the additional collector voltage of the z-MFS increases. This is because, under the condition of increasing additional collector voltage, the electric field of the z-MFS strengthens, making it easier for carriers to flow from the emitter to the collectors and the additional collectors, thereby increasing the current gain of the z-MFS and raising the output voltage.

**Figure 4:**
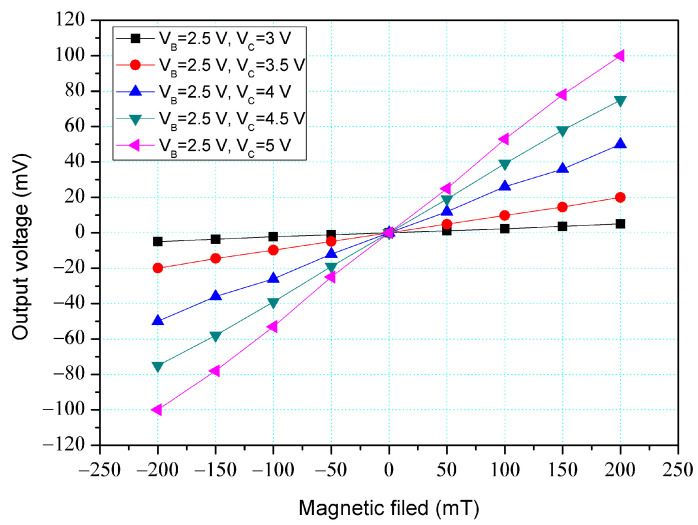
O-V for y-MFS by simulation.

Figure 4 shows the O-V for y-MFS by simulation, where V_B_ is base bias and V_C_ is collector bias. In this evaluation, the collectors linked to a resistance of 1 kΩ. The base bias was 2.5 V. The different voltages of 3, 3.5, 4, 4.5, and 5 V were inputted to the collectors. The results presented that the O-V for the y-MFS increased as the collector bias increased. When the collector and base bias were 5 and 2.5 V, respectively, the O-V for the y-MFS was 100 mV at 200 mT. The use of the linear regression method fits the curve at V_B_ = 2.5 V and V_C_ = 5 V. The regression line had a coefficient of determination R^2^ = 0.9995 and a slope of 510 mV/T. Therefore, the sensitivity of the y-MSE was 510 mV/T at V_B_ = 2.5 V and V_C_ = 5 V, and the output linearity was 99%. The optimal operating point setting for y-MFS was V_B_ = 2.5 V, V_C_ = 5 V, and with the load resistance of 1 K.

**Figure 5:**
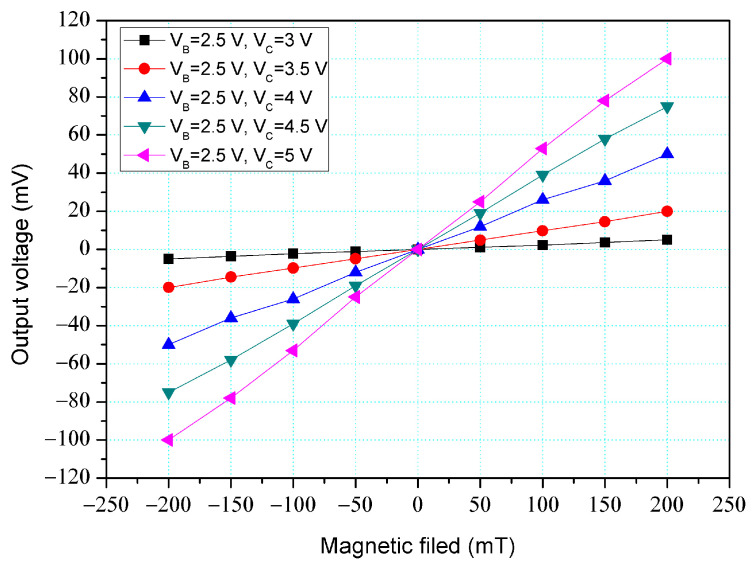
O-V for x-MFS by simulation.

Figure 5 shows the O-V for x-MFS by simulation, where V_B_ is base bias and V_C_ is collector bias. In this evaluation, the collectors linked to a resistance of 1 kΩ. The base (B) bias was 2.5 V. The different voltages of 3, 3.5, 4, 4.5, and 5 V were inputted to the collectors. The results presented that the O-V for the x-MFS increased as the collector bias increased. When the collector and base bias were 5 and 2.5 V, respectively, the O-V for the x-MFS was 100 mV at 200 mT. The use of the linear regression method fits the curve at V_B_ = 2.5 V and V_C_ = 5 V. The regression line had a coefficient of determination R^2^ = 0.9995 and a slope of 510 mV/T. Therefore, the sensitivity of the x-MSE was 510 mV/T at V_B_ = 2.5 V and V_C_ = 5 V, and the output linearity was 99%. The optimal operating point setting for x-MFS was V_B_ = 2.5 V, V_C_ = 5 V, and with the load resistance of 1 K. As shown in Figure 4 and Figure 5, the output voltage of the y-MFS and x-MFS increases with the collector voltage. This is because the electric field in the y-MFS and x-MFS strengthens with an increasing collector voltage, making it easier for carriers to flow from the emitters to the collectors, thus increasing the current gain of the y-MFS and x-MFS and enhancing their output voltage.

**Figure 6:**
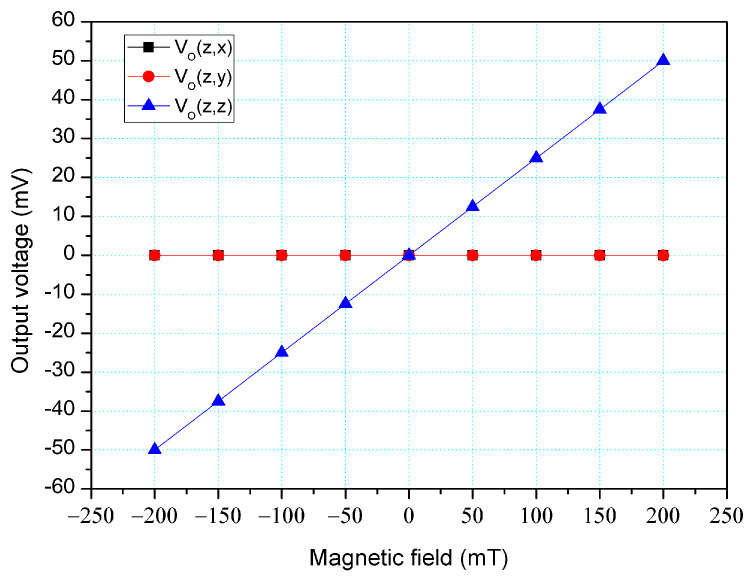
Output for MFS in the z-direction M-F (magnetic field).

**Figure 7:**
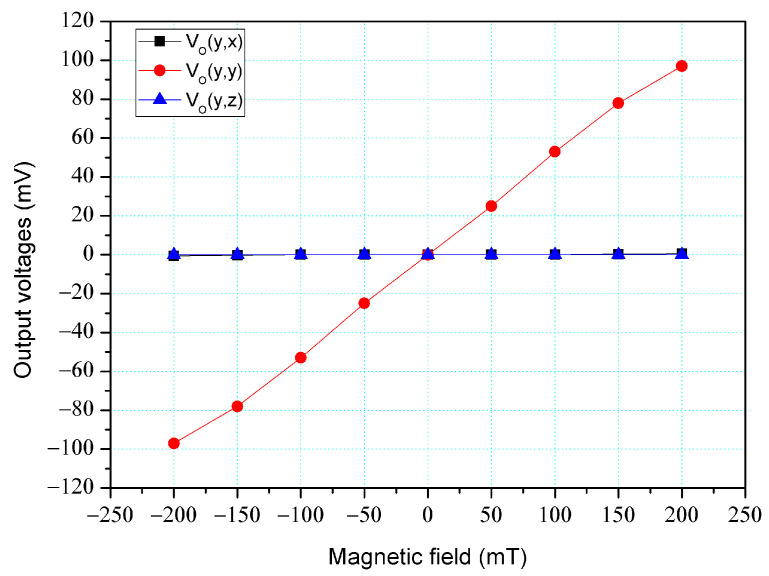
Output for MFS in the y-direction M-F.

**Figure 8:**
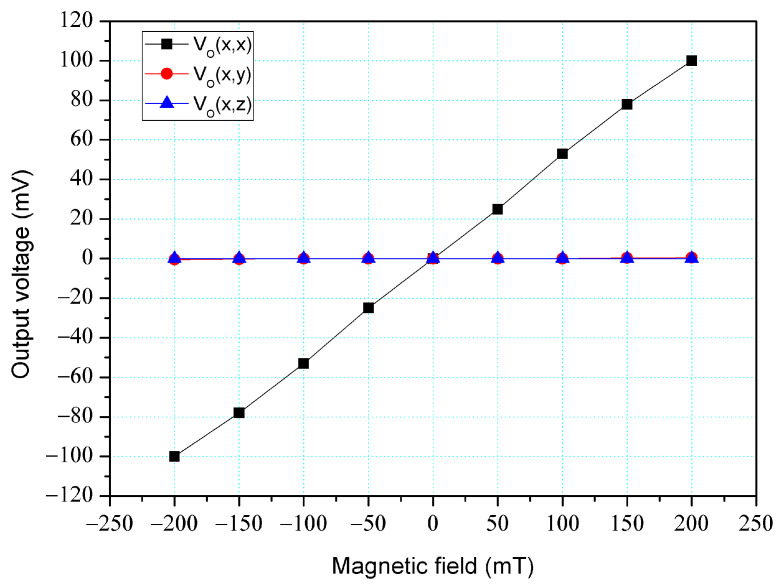
Output for MFS in the x-direction M-F.

The simulation software, Sentaurus TCAD, was also utilized to analyze the cross-sensitivity of the MFS. First, an M-F in the z-direction was applied to the MFS, and the O-V for the z-, y-, and x-MFS was analyzed. Figure 6 shows the analyzed O-V for the x-MFS, y-MFS, and z-MFS in the z-direction M-F, where V_o_(z, x) is the O-V for x-MFS in the z-direction M-F; V_o_(z, y) is the O-V for y-MFS in the z-direction M-F; V_o_(z,z) is the O-V for z-MFS in the z-direction M-F. The O-V of V_o_(z, x) is 0.1 mV at 200 mT and the O-V of V_o_(z,y) is 0.1 mV at 200 mT. This means that the x-MFS and y-MFS have an extremely low response in the z-direction M-F. It is only V_o_(z, z) that shows a high value, which the means that the only z-MFS has a good response in the z-direction M-F. The results depict that the MFS has an extremely low cross-sensitivity in the z-direction M-F. Then, an M-F in the y-direction was applied to the MFS, and the O-V for the z-, y- and x-MFS was analyzed. Figure 7 shows the analyzed O-V for the x-MFS, y-MFS, and z-MFS in the y-direction M-F, where V_o_(y, x) is the O-V for x-MFS in the y-direction M-F; V_o_(y, y) is the O-V for y-MFS in the y-direction M-F; V_o_(y, z) is the O-V for z-MFS in the y-direction M-F. The O-V of V_o_(y, x) is 0.5 mV at 200 mT and the O-V of V_o_(y, z) is 0.1 mV at 200 mT. This means that the x-MFS and z-MFS have an extremely low response in the y-direction M-F. It Is only V_o_(y, y) that shows a high value, which the means that the only y-MFS has a good response in the y-direction M-F. The results present that the MFS has an extremely low cross-sensitivity in the y-direction M-F. Finally, an M-F in the x-direction was applied to the MFS, and the O-V for the z-, y-, and x-MFS was analyzed. Figure 8 shows the analyzed O-V for the x-MFS, y-MFS and z-MFS in the x-direction M-F, where V_o_(x, x) is the O-V for x-MFS in the x-direction M-F; V_o_(x, y) is the O-V for y-MFS in the x-direction M-F; V_o_(x, z) is the O-V for z-MFS in the x-direction M-F. The O-V of V_o_(x, y) is 0.5 mV at 200 mT and the O-V of V_o_(x, z) is 0.1 mV at 200 mT. This means that the y-MFS and z-MFS have an extremely low response in the x-direction M-F. It is only V_o_(x, x) that shows a high value, which means that the only x-MFS has a good response in the x-direction M-F. The results display that the MFS has an extremely low cross-sensitivity in the x-direction M-F. As shown in Figure 7 and Figure 8, x-MFS and y-MFS exhibit nonlinear output voltage under the influence of magnetic fields due to changes in the direction and speed of carrier movement in the MFS caused by the magnetic field, which in turn affects the current gain of the MFS. Therefore, at high magnetic fields, the current gain of the MFS gradually saturates or decreases with the increase of magnetic field, resulting in nonlinear changes in output voltage.

The resolution of a magnetic field sensor can be estimated using the noise level spectrum. This parameter, denoted as *S_B_*, is commonly used to represent magnetic field noise spectral density, and has units of T/$\sqrt{\mathrm{Hz}}$. The *S_B_* of the magnetic field sensor is given by [40]:

(1) $$S_{B}=\frac{S_{V}}{S}$$

where *S_V_* represents the voltage noise spectral density of the sensor with units of V/$\sqrt{\mathrm{Hz}}$, and *S* represents the sensitivity of the sensor with units of V/T. In cases where the magnetic shielding is good, the voltage noise of the signal processing device can be neglected, and *S_B_* is determined by the characteristics of the front-end physical sensing element. Therefore, the resolution of the magnetic field sensor can also be expressed in terms of the root mean square magnetic field noise *B_min_*, with units of Tesla. This parameter can be calculated by [40,41]:

(2) $$B_{min}=\frac{V_{n}}{S}$$

where *V_n_* represents the root mean square voltage noise with units of V and is obtained by integrating the voltage noise spectral density *S_V_* squared with respect to frequency *f*, as follows [40]:

(3) $$V_{n}=\sqrt{\int S_{V}^{2}df}$$

It can be observed from Equation (2) that the resolution of the sensor depends on both the noise level and sensitivity of the sensor.

## 3. Fabrication of Magnetic Field Sensor 

**Figure 9:**
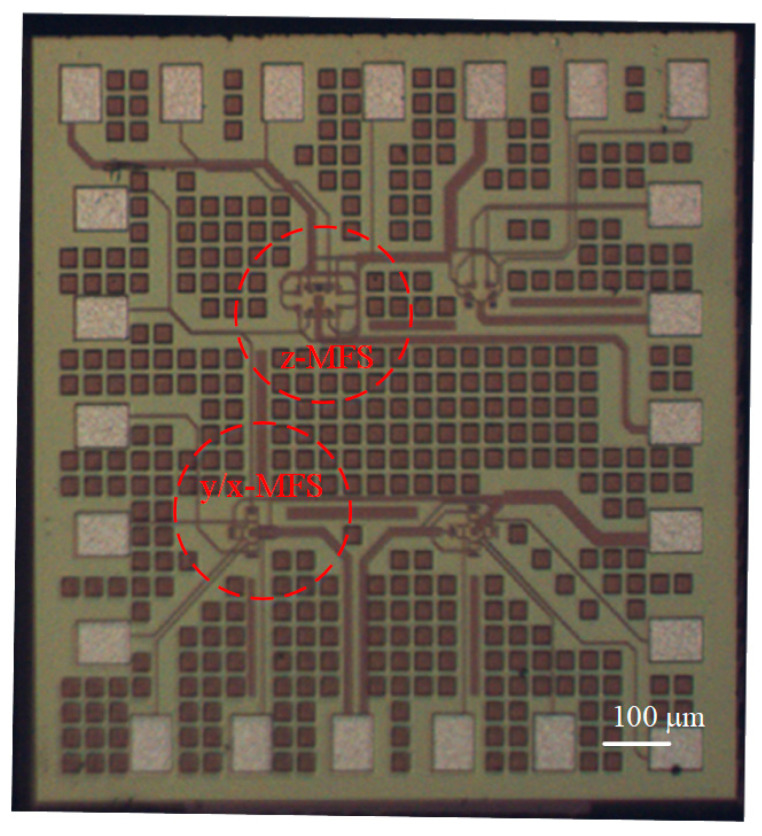
MFS chip photo image.

**Figure 10:**
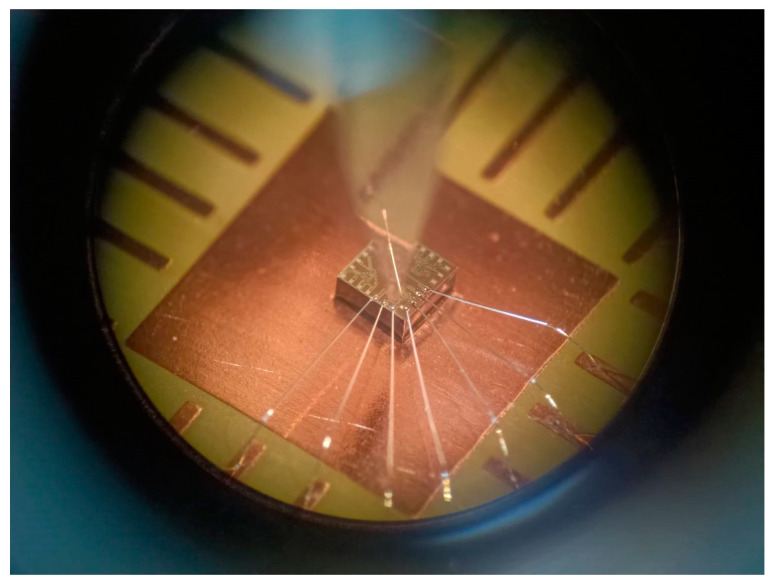
Image of the wire-bonded MS chip.

The MFS chip consisted of z-MFS, y-MFS, and x-MFS, where y-MFS and x-MFS were combined as a y/x-MFS. The z-MFS structure was illustrated in Figure 1. The use of n-well layer limited the moving of current and prevents current leakage. The collectors (C_z1_, C_z2_, C_z3_, and C_z4_), additional collectors (AC_z1_, AC_z2_, AC_z3_, and AC_z4_), and emitter (E_z_) were n-type silicon doping phosphorus [28]. The bases (B_z1_, B_z2_, B_z3_, and B_z4_) were p-type silicon doping boron. The y/x-MFS structure is illustrated in Figure 2. The collectors (C_x1_, C_x2_, C_y1_ and C_y2_) and emitter (E_x1_, E_x2_, E_y1_, and E_y2_) were composed of n-type silicon doping phosphorus. The base (B) was made of p-type silicon doping boron. The z-MFS and y/x-MFS layout were designed according to the structures (Figure 1 and Figure 2). The Taiwan Semiconductor Manufacturing Company (TSMC) carried out the fabrication of the MFS chip using the 1P6M 0.18 μm CMOS process [42]. Figure 9 shows a three-axis MFS chip photo image after completion of the CMOS process. It is obvious that the MFS chip contains a z-MFS and a y/x-MFS. The area of the MFS chip was 1 mm^2^. In order to test the characteristic of the MFS, a wire-bonder was utilized to bond the MFS chip on a printed circuit board. Figure 10 displays an image of wire bonding for the MFS chip.

## 4. Results

**Figure 11:**
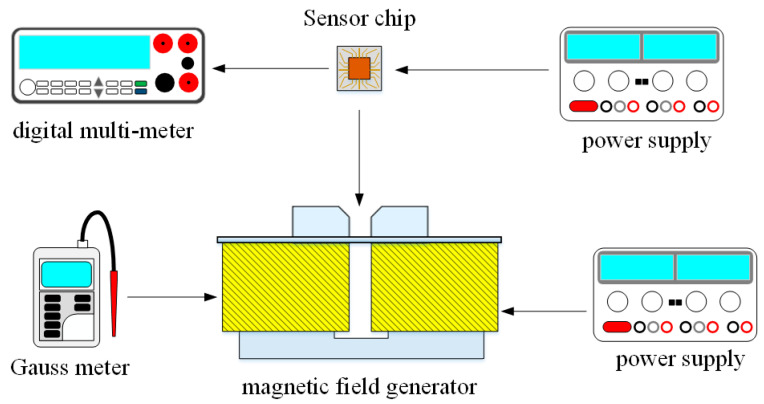
Measurement setup.

The measurement setup for the three-axis MFS was displayed in Figure 11. It contained a Gauss meter (GM08-1029, Hirst, Falmouth, UK), a M-F generator (developed by our lab), a digital multimeter (34405A, Agilent, Santa Clara, CA, USA), and two power supplies. The magnetic field generator was a direct current (DC) type and produced a magnetic field in the range from −250 mT to 250 mT. The Gauss meter was used to calibrate the magnitude of magnetic field produced by the generator. The MFS chip was set on the M-F generator. A power supply provided a power to the M-F generator, and another power supply gave a power to the MFS chip. The M-F generator produced an M-F to the MFS chip. The O-V of the MFS was recorded utilizing the digital multimeter. The measurements were taken inside shielding units. It is possible that some signal loss may occur when using cables or operating in an open environment due to electromagnetic interference. However, we employed proper shielding and grounding techniques to minimize this effect.

**Figure 12:**
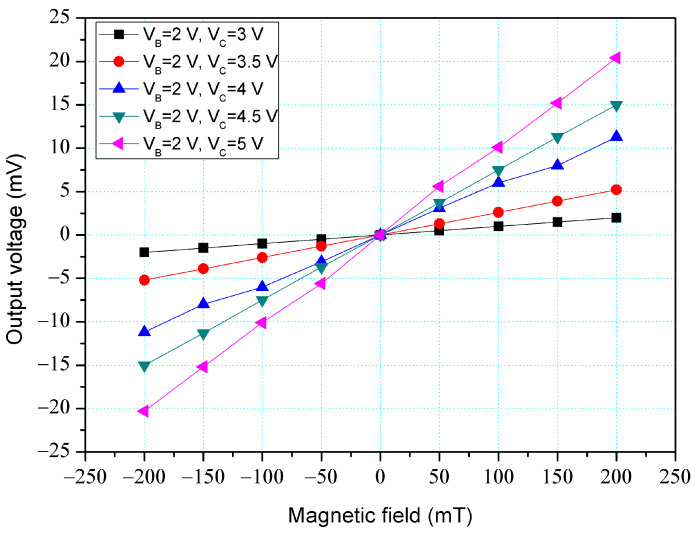
Measurement for z-MFS without the additional collector bias.

The z-MFS performance was measured. As shown in Figure 11, the power supply gave a voltage of 2 V to the base and the different voltages of 3, 3.5, 4, 4.5 and 5 V to the collectors. There was no bias at the additional collectors. The M-F generator gave an M-F of z-direction to the z-MFS. Figure 12 shows the measurement for the z-MFS without the bias of additional collectors, where V_B_ is base bias and V_C_ is collector bias. At the curve of V_B_ = 2 V and V_C_ = 3 V, the O-V of z-MFS was −2.1 mV at −200 mT and 2.2 mV at 200 mT. At the curve of V_B_ = 2 V and V_C_ = 4 V, the O-V of z-MFS was −11.1 mV at −200 mT and 11.2 mV at 200 mT. At the curve of V_B_ = 2 V and V_C_ = 5 V, the O-V of z-MFS was −20.3 mV at −200 mT and 20.4 mV at 200 mT, and the curve had a slope of 101 mV/T. Therefore, the sensitivity for the z-MFS was 101 mV/T when V_B_ = 2 V and V_C_ =5 V. 

**Figure 13:**
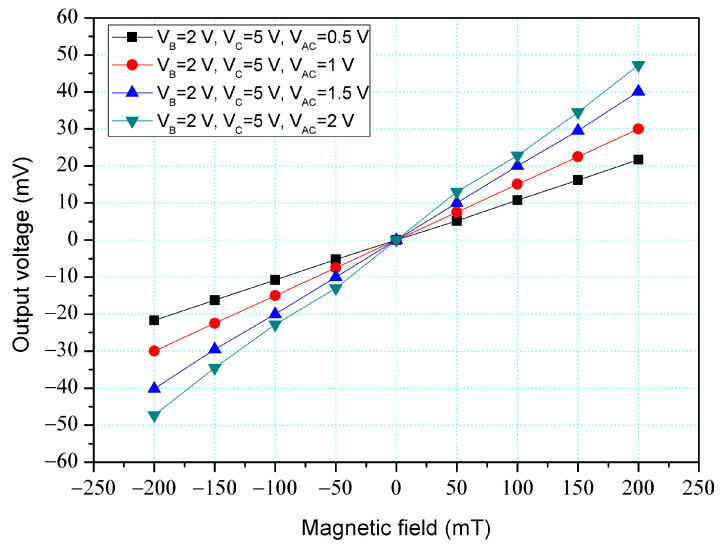
Measurement for z-MFS with the additional collector bias.

In order to increase the z-MFS sensitivity, the z-MFS structure contained the additional collectors. To understand the additional collector effect, the z-MFS was with the additional collector bias measured. As shown in Figure 11, the power supply gave a voltage of 2 V to the base and a voltage of 5 V to the collectors, and the different voltages of 0.5, 1, 1.5, and 2 V were applied to the additional collectors. The M-F generator gave an M-F in the z-direction to the z-MFS. Figure 13 shows the measurement for the z-MFS with the bias of additional collectors, where V_B_ is base bias, V_C_ is collector bias, and V_AC_ is additional collector. At the curve of V_B_ = 2 V, V_C_ = 5 V, and V_AC_ = 0.5 V, the O-V of z-MFS was −21.6 mV at −200 mT and 21.7 mV at 200 mT. At the curve of V_B_ = 2 V, V_C_ = 5 V, and V_AC_ = 1 V, the O-V of z-MFS was −30 mV at −200 mT, and 30.1 mV at 200 mT. At the curve of V_B_ = 2 V, V_C_ = 5 V, and V_AC_ = 2 V, the O-V of z-MFS was −47.2 mV at −200 mT and 47.3 mV at 200 mT, and the curve had a slope of 237 mV/T. So, the sensitivity for the z-MFS was 237 mV/T when V_B_ = 2 V, V_C_ = 5 V, and V_AC_ = 2 V. The power consumption of the z-MFS was 8 mW. Comparing the z-MFS with and without the additional collector bias, the sensitivity of the z-MFS without the additional collector bias was 101 mV/T (at V_B_ = 2 V and V_C_ =5 V in Figure 12), and the sensitivity of the z-MFS with the additional collector bias was 237 mV/T (at V_B_ = 2 V, V_C_ = 5 V and V_AC_ = 2 V in Figure 13). The bias of additional collectors enhanced the sensitivity of the z-MFS. As depicted in Figure 3, the simulated sensitivity of the z-MFS is 250 mV/T. When comparing the simulated sensitivity with the measured one, there was an error percentage of 5.2%, which could be attributed to variations in the doping concentration during the fabrication process of the z-MFS.

**Figure 14:**
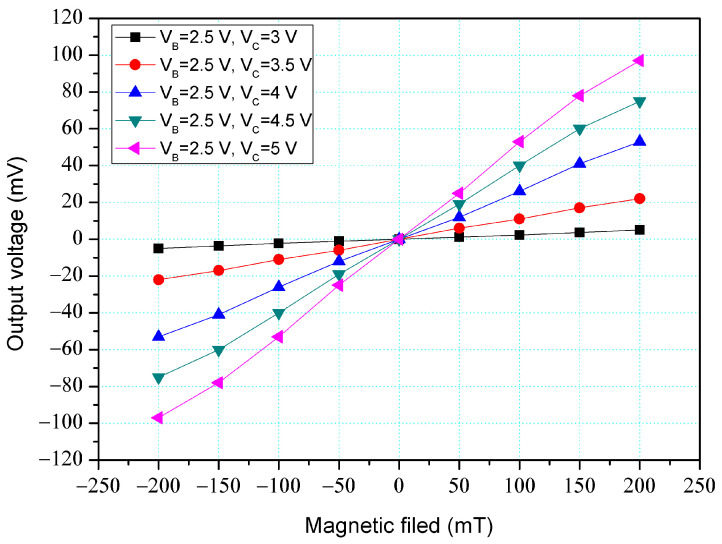
Measurement for y-MFS.

The y-MFS performance was measured. As shown in Figure 11, the power supply gave a voltage of 2.5 V to the base of the y-MFS and the different voltages of 3, 3.5, 4, 4.5, and 5 V to the collectors of the y-MFS. The M-F generator gave an M-F in the y-direction to the y-MFS. Figure 14 shows the measurement for the y-MFS, where V_B_ is base bias and V_C_ is collector bias. At the curve of V_B_ = 2 V and V_C_ = 3 V, the O-V of y-MFS was −5 mV at −200 mT and 5 mV at 200 mT. At the curve of V_B_ = 2 V and V_C_ = 4 V, the O-V of y-MFS was −52.8 mV at −200 mT and 53 mV at 200 mT. At the curve of V_B_ = 2 V and V_C_ = 5 V, the O-V of y-MFS was −96.7 mV at −200 mT and 96.8 mV at 200 mT, and the curve had a slope of 484 mV/T. Therefore, the sensitivity for the y-MFS was 484 mV/T when V_B_ = 2 V and V_C_ = 5 V. The power consumption of the y-MFS was 6 mW. As shown in Figure 4, the simulated sensitivity of the y-MFS is 510 mV/T. The measured sensitivity of the y-MFS had an error percentage of 5.1% compared to the simulation results, which could be attributed to variations in doping concentration during the fabrication process of the y-MFS.

**Figure 15:**
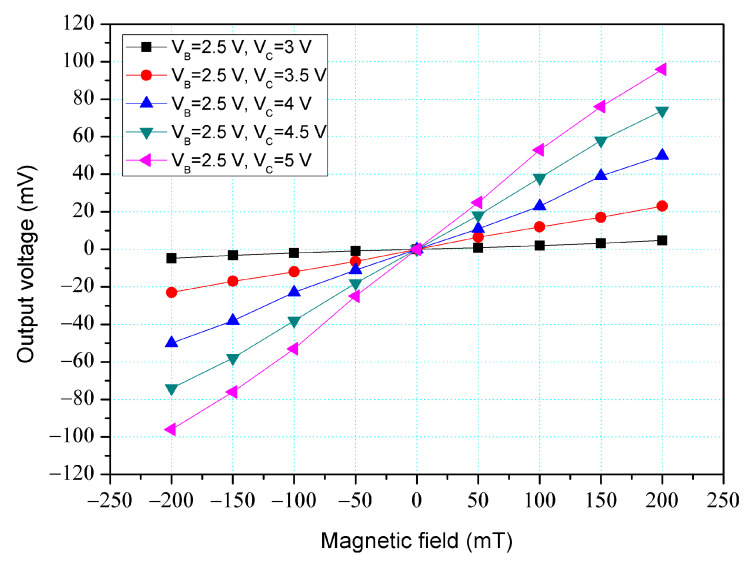
Measurement for x-MFS.

The x-MFS performance was measured. As shown in Figure 11, the power supply gave a voltage of 2.5 V to the base of the x-MFS and the different voltages of 3, 3.5, 4, 4.5, and 5 V to the collectors of the x-MFS. The M-F generator gave an M-F in the x-direction to the x-MFS. Figure 15 shows the measurement for the x-MFS, where V_B_ is the base bias and V_C_ is the collector bias. At the curve of V_B_ = 2 V and V_C_ = 3 V, the O-V of x-MFS was −5 mV at −200 mT and 5.1 mV at 200 mT. At the curve of V_B_ = 2 V and V_C_ = 4 V, the O-V of x-MFS was −53.1 mV at −200 mT and 53.2 mV at 200 mT. At the curve of V_B_ = 2 V and V_C_ = 5 V, the O-V of x-MFS was −96.9 mV at −200 mT and 97 mV at 200 mT, and the curve had a slope of 485 mV/T. So, the sensitivity for the x-MFS was 485 mV/T when V_B_ = 2 V and V_C_ = 5 V. The power consumption of the x-MFS was 6 mW. As shown in Figure 5, the simulated sensitivity of the y-MFS is 510 mV/T. The measured sensitivity of the x-MFS had an error percentage of 4.9% compared to the simulation results, which could be attributed to variations in doping concentration during the fabrication process of the x-MFS.

To characterize the resolution and noise level of the MFS, a Spectrum Analyzer (Advantest R3273) was used to measure the voltage noise of the x-, y-, and z-MFS. The spectrum analyzer displayed the voltage noise spectral density *S_V_* of the MFS. The root mean square voltage noise *V_n_* at the MFS was calculated using Equation (3). The voltage noise spectral density *S_V_* of the x-, y-, and z-MFS was measured, and the respective root mean square voltage noise *V_n_* was evaluated. The results showed that the x-MFS had a *V_n_* of 0.61 µV, the y-MFS had a *V_n_* of 0.58 µV, and the z-MFS had a *V_n_* of 0.26 µV. The sensitivities of the x-, y-, and z-MFS were 484 mV/T, 485 mV/T, and 237 mV/T, respectively. By substituting the *V_n_* value and sensitivity of the x-, y-, and z-MFS into Equation (2), the resolutions of the x-, y-, and z-MFS could be obtained. The resolution of the x-MFS was 1.3 µT, while the resolutions of the y-MFS and z-MFS were 1.2 µT and 1.1 µT, respectively.

**Figure 16:**
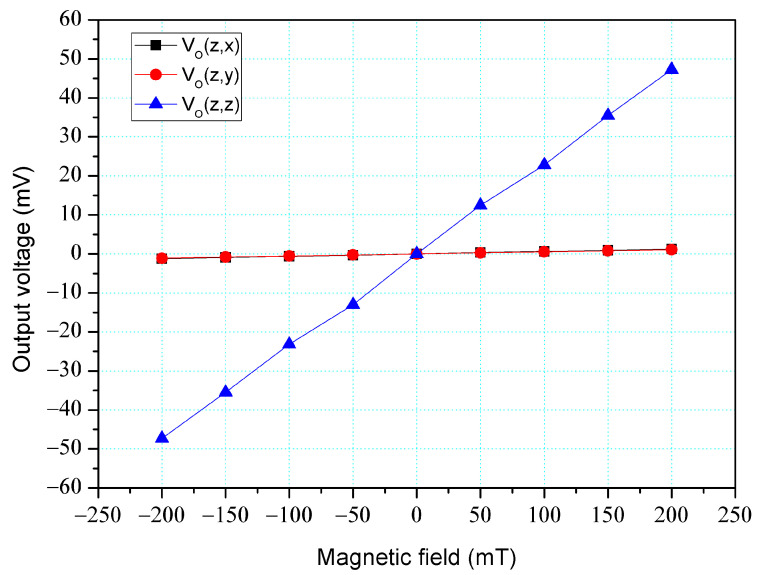
Measurement of MFS under M-F in the z-direction.

The cross-sensitivity is one of important characteristic for the three-axis MFS. To character the MFS cross-sensitivity, the x-, y- and z-MFS were tested under the same direction M-F. As shown in Figure 11, the power supply gave a voltage of 2.5 V to the base of the x-MFS and y-MFS and a voltage of 5 V to the collector of the x-MFS and y-MFS. At the same time, the power supply gave a voltage of 2 V to the bases of the z-MFS, a voltage of 5 to the collectors of the z-MFS and a voltage of 2 V to the additional collectors of the z-MFS. The M-F generator suppled an M-F in the z-direction to the MFS, and the digital multimeter detected the output for the z-MFS, y-MFS, and x-MFS. Figure 16 depicts the measurement of the z-MFS, y-MFS, and x-MFS in the z-direction M-F, where V_o_ (z, x) is O-V for x-MFS in the z-direction M-F; V_o_ (z, y) is O-V for y-MFS in the z-direction M-F; V_o_ (z, z) is O-V for z-MFS in the z-direction M-F. The curve of V_o_ (z, x) had a slope of 6.3 mV/T, and the curve of V_o_ (z, y) had a slope of 6.1 mV/T. The curve of V_o_ (z, z) had a slope of 237 mV/T. This means that the x-MFS had a sensitivity of 6.3 mV/T and the y-MFS had a sensitivity of 6.1 mV/T in the z-direction M-F. The sensitivity of the z-MFS was 237 mV/T, so the MFS had a cross-sensitivity less than 3% in the z-direction M-F. As depicted in Figure 6, the simulated cross-sensitivity of the z-MFS was found to be less than 1%. Furthermore, the difference between the simulated and measured cross-sensitivity values was negligible.

**Figure 17:**
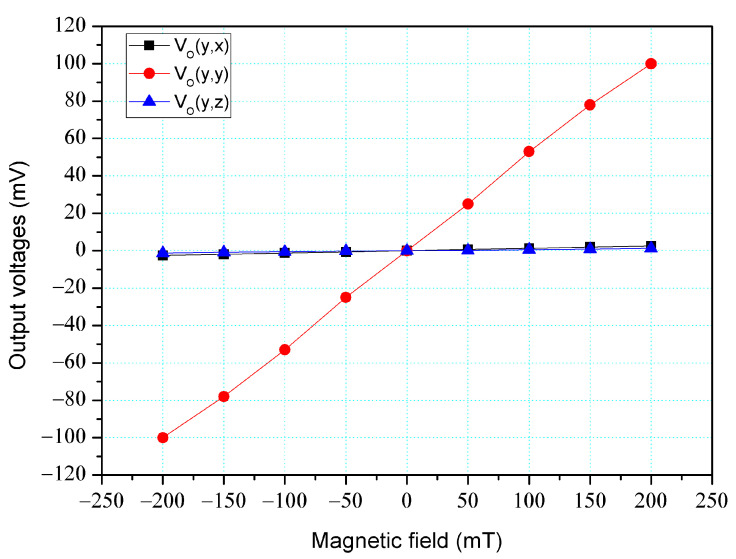
Measurement of MFS under M-F in the y-direction.

The cross-sensitivity of the MFS in the y-direction M-F was measured. As shown in Figure 11, the power supply gave a voltage of 2 V to the bases of the z-MFS, a voltage of 5 to the collectors of the z-MFS and a voltage of 2 V to the additional collectors of the z-MFS. At the same time, the power supply gave a voltage of 2.5 V to the base of the x-MFS and y-MFS and a voltage of 5 V to the collector of the x-MFS and y-MFS. The M-F generator suppled an M-F in the y-direction to the MFS, and the digital multimeter measured the output for the z-MFS, y-MFS, and x-MFS. Figure 17 depicts the measurement of the z-MFS, y-MFS, and x-MFS in the y-direction M-F, where V_o_ (y, x) is O-V for x-MFS in the y-direction M-F; V_o_ (y, y) is O-V for y-MFS in the y-direction M-F; V_o_ (y, z) is O-V for z-MFS in the y-direction M-F. The curve of V_o_ (y, x) had a slope of 12.7 mV/T, and the curve of V_o_ (y, z) had a slope of 5.8 mV/T. The curve of V_o_ (y, y) had a slope of 485 mV/T. This means that the x-MFS had a sensitivity of 12.7 mV/T and the z-MFS had a sensitivity of 5.8 mV/T in the y-direction M-F. The sensitivity of the y-MFS was 485 mV/T, so the MFS had a cross-sensitivity less than 3% in the y-direction M-F. As shown in Figure 7, the simulated cross-sensitivity of the y-MFS was found to be less than 1%. Furthermore, the difference between the simulated and measured cross-sensitivity values was negligible.

**Figure 18:**
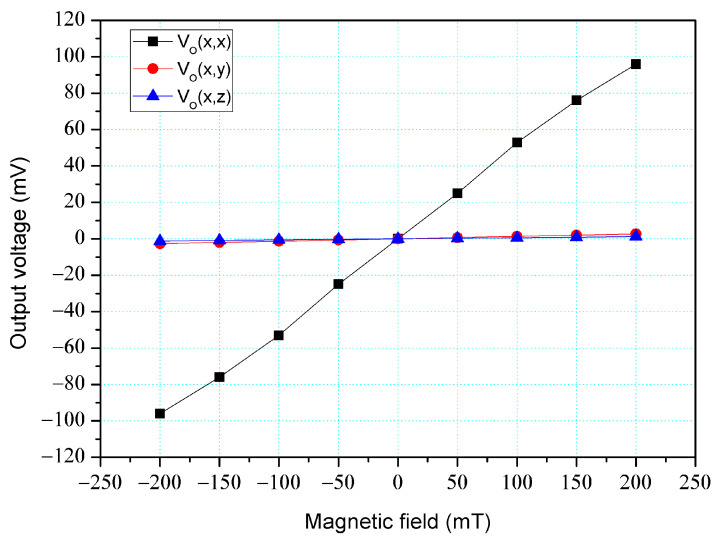
Measurement of MFS under M-F in the x-direction.

The cross-sensitivity of the MFS in the x-direction M-F was tested. As shown in Figure 11, the power supply gave a voltage of 2.5 V to the base of the x-MFS and y-MFS and a voltage of 5 V to the collector of the x-MFS and y-MFS. At the same time, the power supply gave a voltage of 2 V to the bases of the z-MFS, a voltage of 5 to the collectors of the z-MFS, and a voltage of 2 V to the additional collectors of the z-MFS. The M-F generator provided an M-F in the x-direction to the MFS, and the digital multimeter recorded the output for the z-MFS, y-MFS, and x-MFS. Figure 18 depicts the measurement of the z-MFS, y-MFS, and x-MFS in the x-direction M-F, where V_o_ (x, x) is O-V for x-MFS in the x-direction M-F; V_o_ (x, y) is O-V for y-MFS in the x-direction M-F; V_o_ (x, z) is O-V for z-MFS in the x-direction M-F. The curve of V_o_ (x, y) had a slope of 13.1 mV/T, and the curve of V_o_ (x, z) had a slope of 6 mV/T. The curve of V_o_ (z, z) had a slope of 484 mV/T. This means that the y-MFS had a sensitivity of 13.1 mV/T and the z-MFS had a sensitivity of 6 mV/T in the x-direction M-F. The sensitivity of the x-MFS was 484 mV/T, so the MFS had a cross-sensitivity less than 3% in the x-direction M-F. As presented in Figure 8, the simulated cross-sensitivity of the x-MFS was found to be less than 1%. Furthermore, the difference between the simulated and measured cross-sensitivity values was negligible.

Table 1 summarizes the sensitivity of various MFSs. The sensors presented by Zhao [24] and Yang [25] are two-axis MFSs, while the sensors developed by Zhao [26], Chen [27], and Wu [28] are three-axis MFSs. In this study, the sensitivities of the z-, y-, and x-axes of the MFS are 237 mV/T, 485 mV/T, and 484 mV/T, respectively. When comparing these sensors [24,25,26,27,28] to the MFS developed in this study, the sensitivity of our MFS exceeds that of the previously mentioned MFSs [24,25,26,27]. The specifications of three market three-axis magnetic field sensors are summarized as follows: (a) The Bosch BMC150 sensor has a resolution of 0.3 µT, a noise level of 1 µT (rms) and a power consumption of 2.6 mW. (b) The Honeywell HMC1001 sensor has a resolution of 2.7 nT, a noise density of 29 nV/$\sqrt{\mathrm{Hz}}$, a power consumption of 2.9 mW, and a sensitivity of 160 mV/T. (c) The Honeywell HMC1021 sensor has a resolution of 8.5 nT, a noise density of 48 nV/$\sqrt{\mathrm{Hz}}$, a power consumption of 2.2 mW, and a sensitivity of 50 mV/T. Compared to these market sensors, our sensor has a higher sensitivity than the Honeywell HMC1001 and Honeywell HMC1021 sensors, but its resolution does not exceed those of these sensors. 

To perform magnetic field measurements simultaneously on all three axes, a data acquisition system is required to read and process data from the MFS. The data acquisition system typically consists of an analog-to-digital converter (ADC) and a microcontroller unit (MCU) that converts analog signals to digital signals and store and process them. In future work, we plan to implement a data acquisition system capable of handling multiple data channels simultaneously to ensure accurate and synchronized acquisition.

## 5. Conclusions

The study has implemented the manufacturing and measurement of a three-axis MFS. The structure of the MFS consisted of a z-MFS and a y/x-MFS, where the y/x-MFS contained a y-MFS and an x-MFS. The z-, y-, and x-MFS, which were a magnetic transistor type, were employed to sense M-F in the z-, y-, and x-direction, respectively. The three-axis MFS had a low cross-sensitivity because z- and y/x-MFS were separated from each other on the chip and the z-, y-, and x-MFS had an independent M-F sensing direction. Adding four additional collectors in the z-MFS improved the sensitivity of the z-MFS owing to the additional collectors, which increased the carrier mobility in the silicon substrate. The sensitivity of the MFS was analyzed utilizing the Sentaurus TCAD. The analyzed results depicted that the sensitivities of the z-, y- and x-MFS were 250, 500 and 500 mV/T, respectively. The commercial CMOS process was adopted to make the MFS chip. The MFS could be easily mass-produced because it does not require post-processing, and can also be produced using the TSMC CMOS process. Experiments revealed that the z-MFS had a sensitivity of 237 mV/T, the y-MFS had a sensitivity of 485 mV/T, and the x-MFS had a sensitivity of 484 mV/T. Comparing the results regarding MFS sensitivity, the error percentage of the experimental results for the MFS sensitivity was less than 5%. The measured results presented that the cross-sensitivities of the z-, y-, and x-MFS were less than 3%. Therefore, the three-axis had a high sensitivity and a low cross-sensitivity. Our three-axis MFS has several advantages. Firstly, the MFS has higher gain and sensitivity, making them more suitable for applications that require precise measurements of magnetic fields. Additionally, the MFS is fabricated using semiconductor foundries, which allows for mass-production at a low cost while maintaining high process stability. Furthermore, our sensor has been specifically designed to have higher sensitivity than existing magnetic field sensors [19,20,21,22,23,24,25,26,27,28].

## Figures and Tables

**Table 1:** Summary of sensitivity for various MFSs.

Authors	Sensitivity (mV/T)		
	x-Axis	y-Axis	z-Axis
Zhao [24]	223	218	-
Yang [25]	366	365	-
Zhao [26]	77.5	78.6	77.4
Chen [27]	182	180	27.8
Wu [28]	534	525	119
This work	485	484	237

## Data Availability

Data sharing not applicable.
